# Gender disparities in patients treated with veno-arterial ECMO for cardiogenic shock complicating acute myocardial infarction

**DOI:** 10.3389/fcvm.2025.1461580

**Published:** 2025-05-22

**Authors:** Lingyue Qiu, Yingzhong Lin, Meiying Long, Qingkuan Li, Xiyong Sheng, Ying Shi, Changhua Mo, Qili Huang, Mengjie Wang, Xubin Wu, Ling Liu, Zhengde Lu, Guozheng Qiu, Liwen Lyu, Qingwei Ji

**Affiliations:** ^1^ Department of Cardiology, Guangxi Chest Pain Center, The People’s Hospital of Guangxi Zhuang Autonomous Region, and Institute of Cardiovascular Diseases, Guangxi Academy of Medical Sciences, Nanning, China; ^2^ Department of Emergency, The People’s Hospital of Guangxi Zhuang Autonomous Region and Research Center of Cardiovascular Disease, Guang Xi Academy of Medical Sciences, Nanning, China

**Keywords:** cardiogenic shock, acute myocardial infarction, extracorporeal membrane oxygenation, gender, mortality

## Abstract

**Background:**

It is crucial to investigate whether there are prognostic disparities among AMI-CS patients undergoing VA-ECMO support. However, there is currently limited data available from China.

**Aims:**

To evaluate the gender differences in the characteristics, management, and outcomes among patients with AMI-CS received VA-ECMO support in China.

**Methods:**

Patients admitted with AMI-CS at the Chest Pain Center of Guangxi Zhuang Autonomous Region People's Hospital between 2018 and 2023 were included. Sex differences in baseline characteristics, in-hospital management, and outcomes were compared. The primary endpoint was in-hospital mortality. Propensity score matching (PSM) was performed to reduce the impact of baseline clinical differences. Cox regression analysis was conducted to assess the association between gender and in-hospital mortality.

**Results:**

Among 193 patients presenting with AMI-CS, 15.54% were women. Women were older (67.23 ± 13 vs. 60.37 ± 12.98, *p* = 0.0028), had a higher prevalence of comorbidities, and a lower proportion of smoking history. Women were less likely to receive vascular reconstruction (70% vs. 88%, *p* = 0.023), had a significantly shorter duration of ECMO support (2.72 days vs. 4.87 days, *p* = 0.027), as well as shorter hospitalization days compared to male patients (11.73 ± 10.52 vs. 16.89 ± 10.74, *p* = 0.026). In-hospital all-cause mortality was notably higher among female patients (90.0%) compared to male patients (71.2%, *p* = 0.023). However, after PSM, the difference in in-hospital mortality rates between genders was not statistically significant (*p* = 0.16).

**Conclusion:**

In this retrospective study, women were less likely to receive revascularization and exhibited worse in-hospital outcomes compared to men. However, the observed sex difference in in-hospital mortality was attenuated after adjusting for clinical characteristics and acute treatments among AMI-CS patients receiving ECMO support.

## 1 Introduction

Cardiogenic shock (CS), a life-threatening complication of acute myocardial infarction (AMI), is characterized by the stunned myocardium, significant loss of myocardial tissue and refractory hypotension, making it a leading cause of in-hospital mortality among AMI patients (1). The clinical features of CS complicating AMI (AMI-CS) include persistent or worsening ischemic symptoms, hemodynamic instability, arrhythmias, cardiac arrest, mechanical complications, and acute heart failure. Over the past two decades, the incidence of CS shows an increasing trend worldwide. For instance, analysis of the 2000–2017 National Inpatient Sample databases by Vallabhajosyula revealed a rise in CS complicating ST-Segment Elevation Myocardial Infarction (STEMI-CS) incidence from 5.8% in 2000 to 13.0% in 2017 in the United States (2). Similarly, the China PEACE-Retrospective AMI Study (China Patient-centered Evaluative Assessment of Cardiac Events Retrospective Study of Acute Myocardial Infarction) extracted patients data from 162 hospitals in China, and found that the incidence rate of STEMI-CS was 4.7% in 2001, 5.9% in 2006, and 6.2% in 2011 (3, 4). Despite advancements in reperfusion and pharmacological therapy, in-hospital mortality rates for AMI-CS remain high, ranging between 32% to 66% (2, 5). Early revascularization has been shown to significantly improve short- and long-term prognosis for AMI-CS patients. Additionally, percutaneous mechanical circulatory support, including intra-aortic balloon pump (IABP), extracorporeal membrane oxygenation (ECMO), and Impella, are recommended for AMI-CS patients not responding to standard therapy (6, 7). Recently, veno-arterial ECMO (VA-ECMO) has been increasingly used in AMI-CS due to its availability in China, although the evidence supporting its efficacy remains limited. Therefore, it is crucial to investigate whether there are prognostic disparities among AMI-CS patients undergoing VA-ECMO support.

Numerous studies have demonstrated gender disparities in the characteristics, management, and outcomes of AMI-CS patients (8, 9). Women with AMI-CS tend to be older, present with more comorbidities, acute organ failure, and concomitant cardiac arrest, and receive less coronary angiography, revascularization, and secondary prevention therapies compared to men (10–12). Moreover, randomized controlled trials and cohort studies have consistently reported higher complication rates and lower survival rates among women with AMI-CS, both in the short and long term. However, it remains unclear whether similar gender disparities exist among AMI-CS patients receiving VA-ECMO support. An analysis of the Nationwide Inpatient Sample Database from 2000 to 2014 reported that only 0.5% of AMI-CS patients underwent VA-ECMO treatment, with a high in-hospital mortality rate of 59.2%, and the prognosis of men was worse than that of women (13). Another study using national databases found no significant differences in in-hospital mortality rates between women and men receiving ECMO support, despite women being older and having more comorbidities on average (14). However, results from randomized controlled trials suggest that women may have a poorer prognosis than men with VA-ECMO support (15). For instance, the ECLS-SHOCK randomized trial reported 30-day all-cause mortality rates of 56.4% for women compared to 45.9% for men receiving ECMO support, indicating a gender disparity in this patient population (16). Additionally, a meta-analysis of four randomized controlled trials revealed a mortality rate approximately 10% higher among women, regardless of ECMO support (17). However, there is currently no research examining gender disparities in AMI-CS patients receiving VA-ECMO support in China. Therefore, this study aims to investigate gender disparities in the characteristics, management, and outcomes of AMI-CS patients receiving VA-ECMO support in our center.

## 2 Methods

### 2.1 Study population

This study included patients diagnosed with acute myocardial infarction (AMI) [ST segment elevation MI [STEMI] or non-ST segment elevation MI [NSTEMI]] complicated by cardiogenic shock (CS) at the Chest Pain Center of Guangxi Zhuang Autonomous Region People's Hospital between 2018 and 2023. All patients 18 years or older who presented with AMI complicated by CS, and received VA-ECMO support for refractory CS during their index hospitalization were included in this retrospective analysis. Exclusion criteria were defined as follows: (1) Irreversible central nervous system (CNS) damage, as patients with severe neurological impairment have significantly altered prognoses, which may confound ECMO outcome analysis; (2) Cardiac arrest exceeding 60 min, because prolonged cardiac arrest can result in irreversible organ damage and complicate the analysis of ECMO outcomes; (3) VA-ECMO support following high-risk PCI complications, such as coronary perforation or prolonged cardiac arrest, as these complications introduce bias unrelated to the typical course of AMI-CS; (4) End-stage multiple organ failure, as these patients may have terminal conditions that could affect mortality independently of ECMO therapy; (5) Advanced malignant tumors, due to the impact of terminal cancer on mortality outcomes, which would obscure the effects of AMI-CS and ECMO on mortality; and (6) Incomplete clinical data, to ensure the accuracy and reliability of the analysis. The study was registered at https://www.chictr.org.cn (Unique identifier: ChiCTR2100053009) and has been performed in accordance with the ethical principles of the Helsinki Declaration. The protocol was approved by the ethics committee of People's hospital of Guangxi Zhuang Autonomous Region (Ethics-KY-QT-202103). All patients provided written informed consent.

### 2.2 Data collection

Comprehensive clinical data were collected, encompassing demographics, medical history, and laboratory parameters. In-hospital treatments and interventions, including coronary angiography results, PCI treatment details, intra-aortic balloon pump (IABP) insertion, vasoactive drug usage, and length of hospital stay, were recorded. Secondary prevention medications were defined as the use of aspirin, P2Y12 inhibitors, beta-blockers, ACE inhibitors/ARBs, and statins during hospitalization. Blood purification therapy included hemodialysis (HD) and continuous renal replacement therapy (CRRT), which were initiated based on standard clinical indications for acute kidney injury (AKI). Major in-hospital complications such as gastrointestinal bleeding, infections, abnormal liver function, renal insufficiency, acute heart failure, lower limb thrombosis, and cerebrovascular accidents were also documented.

### 2.3 ECMO management

Veno-arterial ECMO was performed using centrifugal pumps and coated catheters manufactured by Maquet (Germany) or Medtronic (USA). Cannulation of the femoral vein and femoral artery or catheter insertion through surgical incisions was guided by ultrasound. Flow direction was from the femoral vein to the centrifugal pump, then to the extracorporeal membrane lung, and finally to the femoral artery. A distal perfusion catheter was employed in the same-side femoral artery to prevent distal limb ischemia during ECMO support. Systemic anticoagulation with heparin was administered to maintain an activated clotting time (ACT) of 180–200 s, which was continuously monitored and adjusted to ensure effective anticoagulation during ECMO support, minimizing the risk of thromboembolism while avoiding excessive bleeding. The management of ECMO, including anticoagulation and monitoring, was performed by a multidisciplinary team specializing in critical care, with continuous evaluation of the patient's hemodynamics, oxygenation, and overall response to therapy. The indications for initiation and weaning from ECMO are based on the 2020 EACTS/ELSO/STS/AATS expert consensus on post-cardiotomy extracorporeal life support in adult patients (18, 19). VA-ECMO weaning is considered when the ECMO flow rate is reduced to 10%-20% of the normal cardiac output, with no signs of severe cardiac decompensation and stable lactate levels. The patient's requirement for vasopressor support should be minimal, and hemodynamics must remain stable with adequate systemic blood pressure. No significant malignant arrhythmias should occur, and lactate levels should remain stable, with no significant electrolyte imbalances. Additionally, echocardiographic assessment should show a left ventricular ejection time greater than 200 ms and a left ventricular ejection fraction above 40%.

### 2.4 Endpoints

The primary endpoint was in-hospital mortality, defined as all-cause mortality occurring during the patient's hospital stay. Secondary endpoints included major in-hospital complications such as infections, acute heart failure, gastrointestinal bleeding, cerebrovascular accidents, lower limb thrombosis, and liver or kidney dysfunction.

Acute heart failure was defined as the occurrence of low cardiac output syndrome (LCOS) during ECMO support, meeting at least one of the following criteria: an increase in ECMO flow rate (≥0.5 L/min) or the need for additional mechanical circulatory support (e.g., IABP or Impella); persistent lactate elevation (>4 mmol/L) or the requirement for initiation or escalation of inotropic therapy. Infection was defined based on microbiological and clinical criteria, including positive cultures from blood, sputum, urine, or wound secretions, or clinical evidence of infection characterized by fever, leukocytosis or leukopenia, and organ-specific dysfunction (e.g., pneumonia, urinary tract infection, or bloodstream infection). Gastrointestinal bleeding was defined as endoscopic evidence of active bleeding, ulcers, erosions, or vascular lesions, or the presence of melena or hematemesis, accompanied by a hemoglobin decrease of >2 g/dl, after excluding other obvious sources of bleeding. Liver dysfunction was defined as an aspartate aminotransferase (AST) or alanine aminotransferase (ALT) level exceeding three times the upper limit of normal, with or without an associated increase in bilirubin levels. Kidney dysfunction was assessed based on serum creatinine elevation and urine output, with the requirement for renal replacement therapy [e.g., continuous renal replacement therapy (CRRT)] used as a criterion for severe kidney injury.

### 2.5 Statistical analysis

Categorical variables were expressed as percentages (%), while continuous variables were presented as means ± standard deviations. Baseline characteristics were compared using Student's *t*-test or Mann–Whitney *U* test for continuous variables and Chi-square test or Fisher's exact test for categorical variables. Survival curves were plotted using the Kaplan–Meier method, and inter-group comparisons were made using the log-rank test. Propensity score matching (PSM) was employed to reduce potential confounding due to baseline clinical differences between male and female patients. The basic principle of propensity score matching is to use a single score to replace multiple covariates, balancing the distribution of covariates between different gender groups. Logistic regression models were established using age, history of hypertension, history of diabetes, duration of ECMO support, and whether vascular reconstruction was performed as matching factors. The matching was based on clinical factors that were considered to influence the outcomes of AMI-CS patients receiving VA-ECMO support. PS values for different gender subgroups were calculated separately. Female and male patients were matched in a 1:1 ratio based on their estimated PS values using nearest neighbor matching, with a caliper value of 0.4. To assess the independent effect of gender on in-hospital mortality, Cox proportional hazards regression was performed before and after PSM. Univariate Cox regression analysis was conducted to assess the association between gender and in-hospital mortality, while multivariate Cox regression analysis was performed to adjust for confounding variables, estimating hazard ratios (HRs) and 95% confidence intervals (CIs). All statistical analyses were performed using R 4.2.0, with two-sided tests and a significance level set at 0.05.

## 3 Results

### 3.1 Baseline characteristics

**Figure 1:**
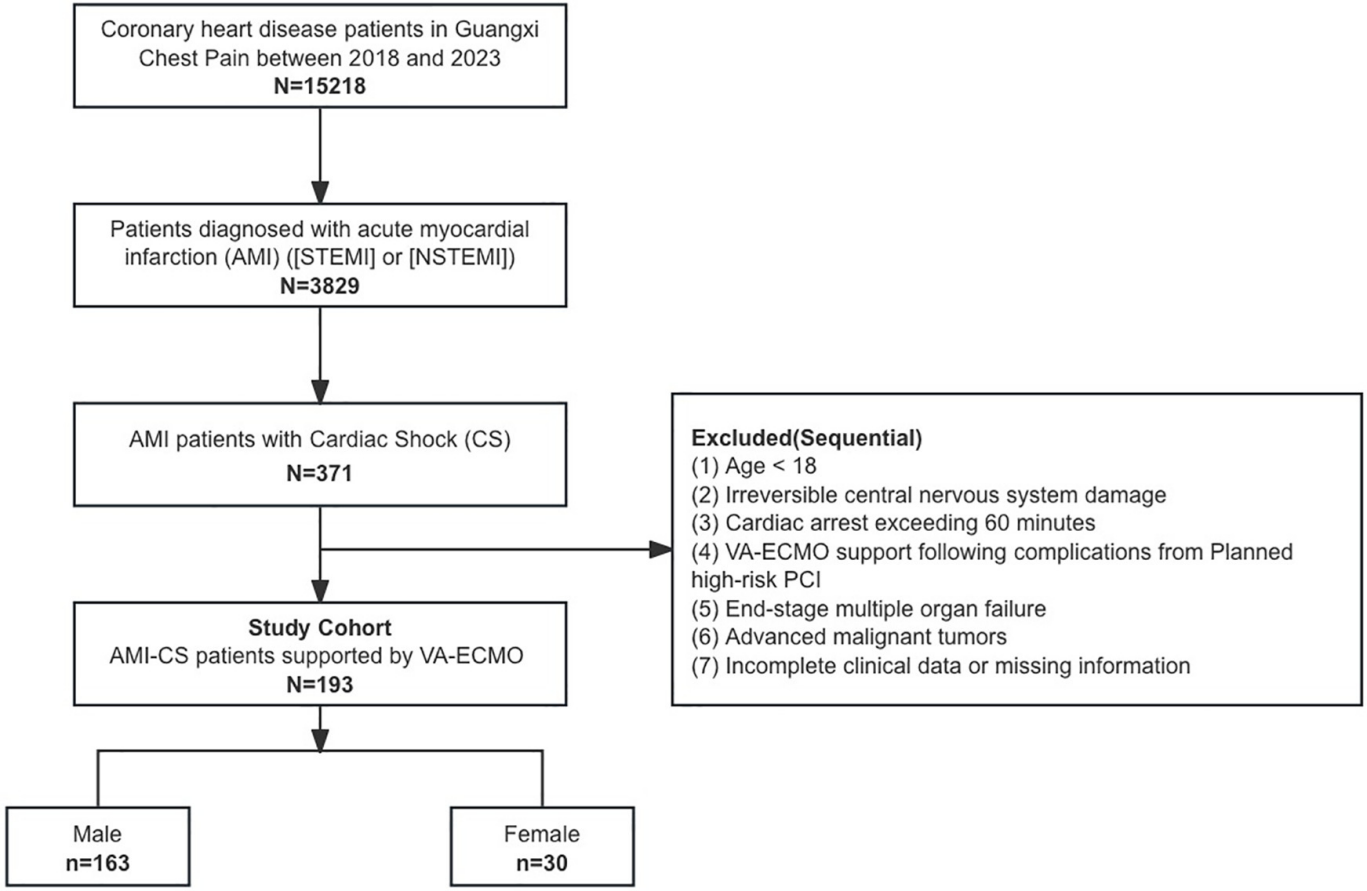
Study protocol.

A total of 193 patients with AMI treated with VA-ECMO were included in the study, comprising 30 females and 163 males (Figure 1). Compared to male patients, female patients were older (67.23 ± 13 vs. 60.37 ± 12.98, *p* = 0.0028), had a higher proportion of comorbidities such as hypertension and diabetes (*p* < 0.05), and a lower proportion of smoking history (*p* < 0.001). 127 (65.8%) patients were transferred from other hospitals, with a higher proportion of male transfers compared to female transfers (69% vs. 50%, *p* = 0.047). Baseline characteristics and laboratory examinations are summarized in Tables 1 and Supplementary Table S1. After propensity score matching, 29 male and 29 female patients were matched, achieving baseline balance between the two groups, except for smoking history, with no significant differences observed in baseline characteristics between male and female patients (*p* > 0.05).

**Table 1:** Baseline characteristics and details of coronary angiography.

Variable	Unmatched group			Matched group		
	Female, *N* = 30^a^	Male, *N* = 163^a^	*p*-value^b^	Female, *N* = 29^a^	Male, *N* = 29^a^	*p*-value^b^
Age (year), mean (SD)	67.23 (13.00)	62.18 (12.00)	0.028	66.97 (13.14)	63.41 (15.68)	0.35
BMI	22.64 (7.32)	23.22 (2.73)	0.77	22.49 (7.59)	22.82 (2.70)	0.88
Smoking, *n* (%)	0 (0%)	52 (32%)	<0.001	0 (0%)	9 (31%)	0.002
Drinking, *n* (%)	5 (17%)	28 (17%)	>0.9	5 (17%)	3 (10%)	0.7
Medical history, *n* (%)						
Hypertension	21 (70%)	81 (50%)	0.041	20 (69%)	18 (62%)	0.6
Diabetes mellitus	15 (50%)	47 (29%)	0.023	14 (48%)	11 (38%)	0.4
Coronary heart disease	3 (10%)	31 (19%)	0.2	2 (6.9%)	4 (14%)	0.7
Myocardial infarction	1 (3.3%)	9 (5.5%)	>0.9	1 (3.4%)	0 (0%)	>0.9
CKD	1 (3.3%)	2 (1.2%)	0.4	1 (3.4%)	0 (0%)	>0.9
Cerebrovascular diseases	2 (6.7%)	11 (6.7%)	>0.9	1 (3.4%)	3 (10%)	0.6
Previous PCI, *n* (%)	2 (6.7%)	21 (13%)	0.5	1 (3.4%)	3 (10%)	0.6
Transfer, *n* (%)	15 (50%)	112 (69%)	0.047	15 (52%)	18 (62%)	0.4
Cardiac arrest, *n* (%)	10 (33%)	83 (51%)	0.076	10 (34%)	16 (55%)	0.11
Coronary angiography and PCI						
Culprit artery			0.2			0.052
LAD	22 (73%)	95 (58%)		21 (72%)	17 (59%)	
LCX	3 (10%)	11 (6.7%)		3 (10%)	1 (3.4%)	
LM	0 (0%)	19 (12%)		0 (0%)	6 (21%)	
RCA	5 (17%)	35 (21%)		5 (17%)	5 (17%)	
Number of vessels diseased			0.82			0.81
1	8 (27%)	35 (21%)		8 (28%)	6 (21%)	
2	7 (23%)	42 (26%)		7 (24%)	7 (24%)	
3	15 (50%)	86 (53%)		14 (48%)	16 (55%)	
PCI	21 (70%)	143 (88%)	0.023	21 (72%)	23 (79%)	0.54
Stent			0.049			0.27
1	7 (64%)	50 (46%)		7 (64%)	8 (40%)	
2	1 (9.1%)	40 (37%)		1 (9.1%)	7 (35%)	
3	2 (18%)	12 (11%)		2 (18%)	3 (15%)	
4	0 (0%)	6 (5.6%)		0 (0%)	2 (10%)	
5	1 (9.1%)	0 (0%)		1 (9.1%)	0 (0%)	

^a^ *n* (%); Mean (SD).

^b^ Pearson's Chi-squared test; Wilcoxon rank sum test; Fisher's exact test.

### 3.2 In-hospital interventions

There was no significant difference in the severity of coronary artery disease between male and female patients. In terms of in-hospital interventions, the proportion of vascular reconstruction was significantly lower in female patients compared to male patients (70% vs. 88%, *p* = 0.023). Female patients had a significantly shorter duration of ECMO support (2.72 days vs. 4.87 days, *p* = 0.027), as well as shorter hospitalization days compared to male patients (11.73 ± 10.52 vs. 16.89 ± 10.74, *p* = 0.026). There were no significant differences between the two groups in the proportions of patients receiving blood purification therapy, blood transfusion, or IABP insertion. The proportions of patients receiving vasoactive drugs and secondary prevention medications for coronary artery disease were also similar between male and female patients. Details of coronary angiography, in-hospital interventions, and medication use for male and female patients are presented in Tables 1–3. Laboratory tests are shown in Supplementary Table S1.

**Table 2:** ECMO and in-hospital management.

Variable	Unmatched group			Matched group		
	Female, *n* = 30^a^	Male, *n* = 163^a^	*p*-value^b^	Female, *n* = 29^a^	Male, *n* = 29^a^	*p*-value^b^
ECMO support time/days	2.72 (0.63, 6.07)	4.81 (1.95, 7.91)	0.027	2.74 (0.62, 6.16)	5.71 (1.39, 8.94)	0.12
Blood transfusion	29 (97%)	158 (97%)	>0.9	28 (97%)	27 (93%)	>0.9
Blood purification	26 (87%)	149 (91%)	0.5	25 (86%)	26 (90%)	>0.9
IABP	8 (27%)	54 (33%)	0.5	8 (28%)	8 (28%)	>0.9
Hospitalization-time	11.73 (10.53)	16.89 (15.14)	0.026	11.52 (10.65)	15.07 (12.91)	0.26

^a^ *n* (%); Median (IQR).

^b^ Pearson's Chi-squared test; Wilcoxon rank sum test; Fisher's exact test.

**Table 3:** Vasoactive drugs and secondary prevention medications.

Variable	Unmatched group			Matched group		
	Female, *n* = 30^a^	Male, *n* = 163^a^	*p*-value^b^	Female, *n* = 29^a^	Male, *n* = 29^a^	*p*-value^b^
Dopamine	29 (97%)	154 (94%)	>0.9	29 (100%)	27 (93%)	0.5
Adrenaline	15 (50%)	90 (55%)	0.6	15 (52%)	21 (72%)	0.1
Isoproterenol	22 (73%)	123 (75%)	0.8	22 (76%)	20 (69%)	0.6
Norepinephrine	16 (53%)	59 (36%)	0.077	16 (55%)	8 (28%)	0.033
Aspirin	25 (83%)	118 (72%)	0.2	24 (83%)	22 (76%)	0.5
Clopidogrel	21 (70%)	130 (80%)	0.2	20 (69%)	23 (79%)	0.4
*β*-blocker	14 (47%)	68 (42%)	0.6	13 (45%)	11 (38%)	0.6
Statin	23 (77%)	131 (80%)	0.6	22 (76%)	22 (76%)	>0.9
ACEI/ARB	7 (23%)	24 (15%)	0.3	6 (21%)	6 (21%)	>0.9
ARNI	5 (17%)	25 (15%)	0.8	5 (17%)	4 (14%)	>0.9

^a^ *n* (%).

^b^ Pearson’s Chi-squared test; Wilcoxon rank sum test; Fisher's exact test.

### 3.3 Major adverse events

**Figure 2:**
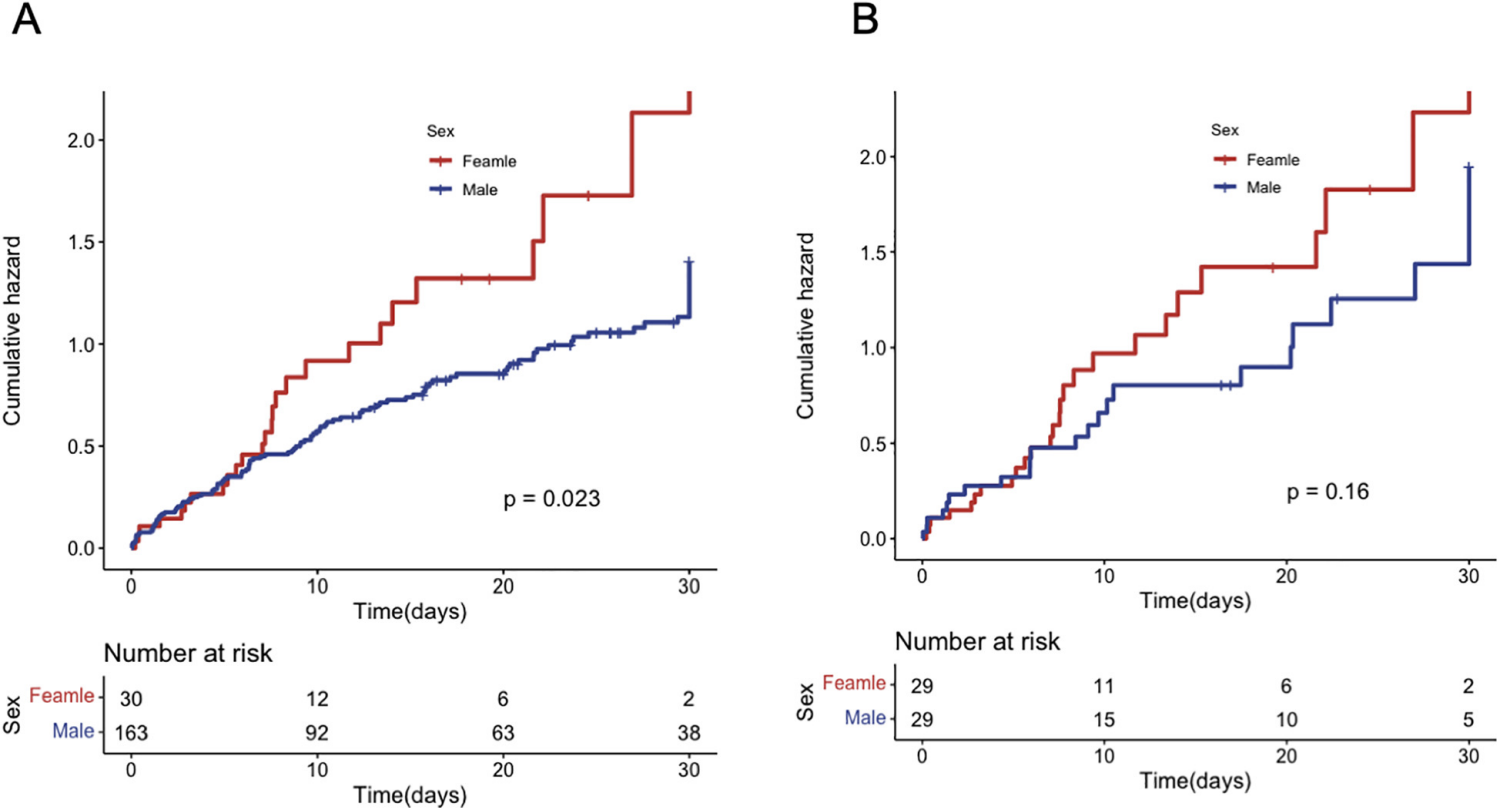
In-hospital mortality for patients of different genders. **(A)** before PS matching; **(B)** after PS matching.

A total of 143 (74.1%) patients died during hospitalization, including 27 (90.0%) females and 116 (71.2%) males. Female patients had a lower incidence of infections but a higher incidence of cerebrovascular accidents (Table 4). Kaplan–Meier analysis showed that the in-hospital all-cause mortality was significantly higher in female patients compared to male patients (*p* = 0.023) (Figure 2A). Univariate Cox regression analysis revealed that female patients had a 63% higher risk of in-hospital mortality compared to male patients (HR = 1.65, 95% CI = 1.08–2.52, *p* = 0.021). After adjustment for multiple factors, female patients had a trend toward higher mortality rate, although the difference was not statistically significant (HR = 1.3, 95% CI = 0.81–2.08, *p* = 0.3). Age and multi-vessel disease were independently associated with mortality, while blood purification therapy and vascular reconstruction significantly improved the prognosis of AMI patients under VA-ECMO support (Table 5). After propensity score matching, Kaplan–Meier analysis showed a trend toward higher in-hospital mortality in female patients, but this difference did not reach statistical significance (*p* = 0.16) (Figure 2B). Results of multivariate Cox regression analysis after propensity score matching are presented in Table 6.

**Table 4:** Major adverse events.

Variable	Unmatched group			Matched group		
	Female, *n* = 30^a^	Male, *n* = 163^a^	*p*-value^b^	Female, *n* = 29^a^	Male, *n* = 29^a^	*p*-value^b^
Death	27 (90%)	116 (71%)	0.03	27 (93%)	23 (79%)	0.3
Hepatic injury	17 (57%)	94 (58%)	>0.9	17 (59%)	17 (59%)	>0.9
Infection	21 (70%)	141 (87%)	0.032	21 (72%)	26 (90%)	0.094
Heart failure	9 (30%)	49 (30%)	>0.9	9 (31%)	6 (21%)	0.4
Arrhythmia	11 (37%)	54 (33%)	0.7	11 (38%)	10 (34%)	0.8
Renal dysfunction	11 (37%)	90 (55%)	0.062	11 (38%)	11 (38%)	>0.9
Gastrointestinal bleeding	8 (27%)	44 (27%)	>0.9	8 (28%)	9 (31%)	0.8
Cerebrovascular diseases	12 (40%)	36 (22%)	0.037	12 (41%)	5 (17%)	0.043
Limb thrombosis	2 (6.7%)	21 (13%)	0.5	2 (6.9%)	3 (10%)	>0.9

^a^ *n* (%); Median (IQR).

^b^ Pearson’s Chi-squared test; Wilcoxon rank sum test; Fisher's exact test.

**Table 5:** Univariate and multivariate COX regression before propensity score matching.

Characteristics	Univariate cox			Multivariate cox		
	HR	95% CI	*p*-value	HR	95% CI	*p*-value
Female	1.65	1.08–2.52	0.021	1.3	0.81, 2.08	0.3
Age	1.02	1.01–1.03	0.007	1.01	1.00, 1.03	0.08
ECMO support time	0.95	0.92–0.99	0.016	0.97	0.94, 1.01	0.2
Blood transfusion	0.8	0.3–2.18	0.668			
Blood purification	0.43	0.25–0.74	0.002	0.55	0.31, 0.98	0.043
Multivessel disease	1.6	1.05–2.44	0.027	1.65	1.08, 2.54	0.022
Cardiac arrest	1.21	0.87–1.68	0.255			
Transfer	0.87	0.61–1.22	0.414			
Medical history						
Coronary heart disease	1.29	0.85–1.97	0.231			
Myocardial infarction	1.42	0.7–2.9	0.335			
PCI	1.2	0.73–1.97	0.471			
Hypertension	1.12	0.81–1.56	0.498			
Diabetes	1.17	0.83–1.66	0.37			
Cerebrovascular disease	0.53	0.25–1.13	0.1			
Smoking	0.6	0.4–0.89	0.011			
Drinking	1.2	0.78–1.85	0.416			
IABP	0.95	0.67–1.35	0.783			

**Table 6:** Univariate and multivariate COX regression after propensity score matching.

Characteristics	Univariate cox			Multivariate cox		
	HR	95% CI	*p*-value	HR	95% CI	*p*-value
Female	1.53	0.87–2.7	0.14	0.84	0.44, 1.60	0.6
Age	1.02	1–1.04	0.041	1.02	0.99, 1.04	0.2
ECMO support time	0.89	0.83–0.96	0.003	0.97	0.94, 1.01	0.2
Blood transfusion	0.14	0.04–0.54	0.004	0.78	0.20, 3.14	0.7
Blood purification	0.2	0.08–0.47	0	0.64	0.24, 1.74	0.4
Multivessel disease	1.53	0.77–3.07	0.228			
Cardiac arrest	1.18	0.68–2.07	0.555			
Transfer	0.59	0.34–1.04	0.069			
Medical history						
Coronary heart disease	1.24	0.53–2.91	0.622			
Myocardial infarction	0	-	0.997			
PCI	0.92	0.33–2.55	0.866			
Hypertension	1.38	0.76–2.5	0.294			
Diabetes	1.08	0.62–1.88	0.796			
Cerebrovascular disease	0.49	0.15–1.6	0.238			
Smoking	0.5	0.21–1.19	0.118			
Drinking	1.62	0.72–3.64	0.245			
IABP	0.89	0.47–1.68	0.724			

## 4 Discussion

In this single-center, retrospective, observational study, we found that women were older, and had a higher prevalence of comorbidities, such as DM and hypertension, compared to male patients. Despite similar access to coronary angiography upon admission, female patients underwent PCI at a significantly lower rate than male patients. Both genders had similar utilization rates of guideline-directed medications. Female patients had a lower proportion of transfers and a shorter duration of ECMO assistance during hospitalisation compared with males. Although female patients experienced fewer infections, and the occurrence of other in-hospital adverse events did not significantly differ from males, in-hospital mortality was markedly higher among female patients. The sex difference in in-hospital mortality was attenuated after adjusting for clinical characteristics and acute treatments in AMI-CS patients receiving ECMO support.

Age is a major determinant of the risk of ACS. Due to the cardioprotective effects of estrogen, the average onset age of ACS is significantly higher in women than in men (20, 21). The results of the CCC-ACS project showed that the mean ages of female and male patients with STEMI were 68.7 ± 10.7 and 59.9 ± 12.4 years, respectively (22). Similarly, in the context of Non-ST-Elevation-ACS (NSTE-ACS), women were approximately six years older on average (69.2 ± 10.5 vs. 63.3 ± 12.0). This age difference between male and female patients also exists in AMI-CS. Analyzing 159,339 patients with STEMI-CS between January 2006 to September 2015 from National Inpatient Sample (NIS) database, Ya'qoub et al. found a significant age discrepancy between female and male patients (age: 69.8 years vs. 63.2 years) across various ethnicities (23–25). Mortality rates increased significantly with increasing age in patients with ACS, irrespective of thrombosis, PCI, coronary artery bypass grafting (CABG),or conservative treatment (26, 27). Numerous studies have demonstrated that the poor prognosis of female patients with ACS can be attributed to a significantly older onset age compared to males. This tendency was also found in CS patients under ECMO support. The international Extracorporeal Life Support Organization (ELSO) registry, analyzing 3,846 cases of ECMO-assisted CS, found a marked age difference between survivors and non-survivors (average age 51 and 56 years, respectively), and created the SAVE-score to predict in-hospital survival in CS patients under VA-ECMO support (16). In SAVE score, the score decreases with age, with 0 points above 63 years old, indicating that the survival of CS may significantly decrease with age. However, the causes of CS in this study are relatively complex, including myocarditis, congenital heart disease, and heart lung transplantation, with acute myocardial infarction accounting for only 29% of patients. The ENCOURAGE mortality score, which factors in age, gender, body mass index, and several clinical parameters, demonstrates superior predictive capability over traditional shock-related risk assessments, emphasizing age as a crucial risk factor (27). In models predicting in-hospital mortality for ECMO-supported AMI patients, an age greater than 65 has been consistently identified as an independent predictive factor. The absence of or unsuccessful vascular reconstruction also markedly increases mortality risk (28). A recent meta-analysis reaffirmed that age above 65 remains an independent predictor of short-term mortality in ECMO-assisted AMI-CS scenarios (29). In our study, the mean age of female patients exceeded 65 years, and was approximately 8 years older than that of male patients, potentially elucidating the observed disparity in outcomes.

Comorbidities play a critical role in both the onset and prognosis of AMI-CS. Diabetes and hypertension are the most common comorbidities of coronary heart disease, and their role in AMI has been investigated widely. A Spanish study using data from the National Health System has indicated that women patients with STEMI-CS tend to be older and more frequently afflicted with hypertension and diabetes (30). Results from the subgroup analyses of SHOCK trial demonstrated that diabetes patients with AMI-CS were more likely to be female, had a history of MI, congestive heart failure, or hypertension, and had renal insufficiency at admission (31). Although revascularization has shown similar benefits in both diabetes and non-diabetes, the study found that diabetes patients received less revascularization. the in-hospital mortality rate of diabetes patients was significantly higher than that of non-diabetes patients. Additionally, this trend has not changed for twenty years. IABP-SHOCK II trial indicated that the longer the follow-up time, the more significant the adverse effect of diabetes on the prognosis. The results showed that 75.8% diabetes patients died, and 62.1% non-diabetes patients died during 6-year follow up (32). Recently, a retrospective study from South Korea found that a strong correlation between in-hospital mortality and both diabetes and hypertension in VA-ECMO-assisted CS patients (33). In our research, the incidence of hypertension and diabetes was notably higher in women than in men. We speculate that this may be related to poor prognosis in women. Our study found that female patients had lower PCI rates despite all having a culprit artery, likely due to gender biases in clinical decision-making. Female patients, being older and having more comorbidities, may have been considered higher risk for PCI, and delayed recognition or treatment initiation could have contributed to more severe conditions at the time of ECMO initiation, leading to higher mortality. The observed separation in Kaplan–Meier curves at 5–10 days, despite the short ECMO duration in female patients, may reflect delayed ECMO initiation, resulting in more severe organ damage and explaining the increased mortality in females. Additionally, post-ECMO management, particularly in females, likely contributed to the rise in mortality, with post-weaning complications, such as organ failure, playing a key role. These findings underscore the need for further research into gender disparities in early revascularization, timing of interventions, and optimal post-ECMO management to ensure equitable access to treatment for all patients.

In the management of AMI-CS, Guideline-Directed Medical Therapy (GDMT) involves a comprehensive approach that includes both pharmacologic treatments and interventional procedures. Vasopressors such as norepinephrine are commonly used to maintain hemodynamic stability, while inotropes like dobutamine help improve myocardial contractility in these patients. Additionally, anticoagulation therapy is employed to prevent thrombotic events. Mechanical circulatory support options like ECMO, IABP, and Impella are considered when pharmacological support is insufficient. Importantly, early revascularization through PCI or CABG has been shown to improve outcomes in AMI-CS patients. The results of our study revealed no significant difference in medication use, including inotropes, vasopressors, and guideline-directed medical therapies, among AMI-CS patients supported by VA-ECMO. Notably, the severity of coronary artery disease did not significantly differ between female and male patients in our cohort. However, the proportion of women receiving reperfusion therapy was lower than that of men, despite its critical importance in improving the prognosis of AMI patients. Early revascularization is essential for improving the prognosis of AMI patients. A time-dependent analysis conducted on Israeli female ACS patients from 2000 to 2016 revealed that an increase in PCI significantly reduced in-hospital mortality for female STEMI patients. Several studies have indicated gender disparities in the administration of reperfusion therapy for AMI patients. The PEACE study, which included AMI patients from 162 hospitals in China, reported higher in-hospital mortality rates among women, who were less likely to receive guideline-recommended treatments, particularly reperfusion therapy (34). Similarly, an analysis of 312,006 AMI patients in Sweden demonstrated that women had significantly lower rates of early reperfusion therapy compared to men, and this disparity remained after adjusting for age. A Canadian study highlighted gender differences in the management of AMI patients, with a notably lower rate of vascular reconstruction among women. Research has shown that early revascularization significantly improves both short-term and long-term outcomes for patients with AMI and CS. Treatment with PCI during hospitalization has been independently associated with a lower risk of in-hospital mortality (30). In our study, the proportion of female patients undergoing vascular reconstruction was lower than that of males, which may be associated with their poorer prognosis.

This investigation acknowledges certain limitations, primarily its single-center design and the small sample size of female participants, potentially leading to biased outcomes. Second, detailed causes of death were not systematically collected in our study, limiting our ability to analyze specific mortality patterns. Future studies should incorporate a more comprehensive data collection strategy to explore mortality causes and their implications more thoroughly. Moreover, this research primarily addresses the association between gender and in-hospital incidents without extending to long-term follow-up. Future studies, preferably multi-center in nature, involving larger populations and extended follow-up durations, are essential for a deeper understanding of gender disparities and their effects on the prognosis of AMICS patients receiving VA-ECMO support.

## 5 Conclusion

Overall, the findings of this study highlight that in the context of VA-ECMO-supported AMICS, female patients not only exhibit greater ages and higher incidences of comorbidities compared to their male counterparts but also maintain a significantly higher mortality risk. However, the sex difference in in-hospital mortality was attenuated after adjusting for clinical characteristics and acute treatments in AMI-CS patients receiving ECMO support. These findings suggest that addressing gender differences in the management of AMI-CS, including equitable access to reperfusion therapy and guideline-directed treatments, may help improve outcomes for female patients.

## Data Availability

The raw data supporting the conclusions of this article will be made available by the authors, without undue reservation.
