# Supplementary material for: Gender disparities in patients treated with veno-arterial ECMO for cardiogenic shock complicating acute myocardial infarction
Source: Front Cardiovasc Med. 2025 May 22;12:1461580. doi: 10.3389/fcvm.2025.1461580 (PMC12142063; doi:10.3389/fcvm.2025.1461580)
Supplement: Supplementary file 1 [file Table1.docx]

**Gender Disparities in Patients Treated with Veno-Arterial ECMO for Cardiogenic Shock Complicating Acute Myocardial Infarction**

**Supplementary materials**

**Supplementary Table S1 Laboratory examinations**

|  | Before PSM | | | After PSM | | |
| --- | --- | --- | --- | --- | --- | --- |
| Variable | Female, n = 30*^1^* | Male, n = 163*^1^* | *p*-value*^2^* | Female, n = 29*^1^* | Male, n = 29*^1^* | *p*-value*^2^* |
| LVEF (%) | 34.00 (27.00, 51.25) | 31.00 (23.00, 43.00) | 0.3 | 32.00 (27.00, 49.00) | 30.50 (27.00, 39.50) | 0.6 |
| WBC count (10^9^/L) | 9.73 (7.53, 11.94) | 13.50 (9.48, 18.23) | 0.003 | 9.74 (8.44, 12.20) | 14.06 (10.66, 18.53) | 0.008 |
| RBC count (10^9^/L) | 3.91 (2.97, 4.09) | 3.91 (3.31, 4.69) | 0.078 | 3.95 (3.11, 4.10) | 3.87 (3.13, 4.79) | 0.3 |
| Platelet count (10^9^/L) | 210.50 (135.50, 257.00) | 181.00 (139.00, 238.00) | 0.7 | 210.00 (133.00, 258.00) | 180.00 (139.00, 296.00) | >0.9 |
| Hemoglobin level (g/L) | 109.00 (79.75, 124.00) | 113.50 (96.50, 136.25) | 0.072 | 109.00 (80.00, 124.00) | 106.00 (89.00, 136.25) | 0.5 |
| ALB (mg/dL) | 34.45 (24.18, 38.95) | 31.60 (26.90, 36.40) | 0.7 | 33.50 (23.70, 38.20) | 30.70 (24.80, 36.70) | 0.6 |
| Lactic acid | 3.60 (2.08, 14.62) | 6.60 (2.30, 12.36) | 0.97 | 3.52 (2.11, 13.62) | 6.39 (2.34, 12.36) | 0.93 |
| TNT_hs(pg/ml) | 1.28 (0.37, 3.52) | 5.63 (1.46, 10.00) | <0.001 | 1.29 (0.47, 3.79) | 4.48 (2.25, 7.89) | 0.022 |
| pro_BNP (pg/ml) | 3,916.00 (1,422.00, 15,334.00) | 5,477.00 (1,875.50, 13,967.00) | 0.5 | 3,806.50 (1,302.88, 15,648.00) | 4,096.00 (1,587.00, 16,421.50) | 0.9 |
| Creatinine level (mmol/L) | 88.50 (76.25, 129.00) | 143.50 (106.00, 224.75) | <0.001 | 88.00 (75.00, 120.00) | 145.00 (107.00, 188.00) | <0.001 |
| UREA (mmol/L) | 7.25 (5.54, 10.99) | 9.12 (6.54, 13.65) | 0.041 | 7.20 (5.50, 10.60) | 9.40 (6.70, 13.10) | 0.066 |
| Uric acid(μmol/L) | 311.00 (284.00, 500.00) | 487.00 (376.00, 602.25) | 0.002 | 306.50 (282.00, 470.75) | 523.00 (406.00, 595.00) | 0.005 |
| Total cholesterol (mmol/L) | 4.62 (3.31, 5.01) | 3.73 (2.94, 4.58) | 0.14 | 4.33 (3.30, 5.05) | 3.03 (2.62, 3.92) | 0.1 |
| Triglyceride (mmol/L) | 1.06 (0.78, 1.53) | 1.27 (0.90, 1.91) | 0.2 | 1.11 (0.80, 1.54) | 1.38 (0.96, 1.92) | 0.2 |
| High-density lipoprotein (mmol/L) | 1.14 (0.91, 1.41) | 0.81 (0.62, 0.98) | <0.001 | 1.07 (0.86, 1.42) | 0.81 (0.65, 0.90) | 0.002 |
| Low-density lipoprotein (mmol/L) | 2.54 (2.09, 3.27) | 2.39 (1.67, 3.02) | 0.3 | 2.50 (2.07, 3.26) | 2.02 (1.47, 2.71) | 0.11 |
| CK_MB (U/L) | 50.50 (24.50, 126.25) | 129.00 (35.00, 320.50) | 0.017 | 55.00 (24.00, 130.00) | 67.00 (39.00, 240.00) | 0.14 |
| DBIL (μmol/L) | 2.55 (1.70, 6.45) | 4.10 (2.60, 9.20) | 0.016 | 2.60 (1.70, 6.50) | 3.60 (2.35, 9.65) | 0.2 |
| IBIL (μmol/L) | 8.10 (5.78, 11.43) | 7.60 (4.90, 12.70) | 0.8 | 8.20 (5.70, 11.60) | 6.50 (5.43, 11.03) | 0.5 |
| *^1^* n (%); Median (IQR) | | | |  |  |  |
| *^2^* Pearson’s Chi-squared test; Wilcoxon rank sum test; Fisher’s exact test | | | |  |  |  |
